# Small Bowel Strangulation in an Adult From an Internal Hernia Caused by a Rare Congenital Mesenteric Defect

**DOI:** 10.7759/cureus.85247

**Published:** 2025-06-02

**Authors:** Tameem Jamal, Saptarshi Biswas

**Affiliations:** 1 General Surgery, Grand Strand Medical Center, Myrtle Beach, USA; 2 Surgery, Grand Strand Medical Center, Myrtle Beach, USA

**Keywords:** acute abdomen, bowel obstruction, congenital transmesenteric defect, internal hernia, ischemic bowel

## Abstract

Internal hernia occurs when a portion of the bowel herniates through a congenital or acquired opening within the abdominal cavity. This can lead to small bowel obstruction, bowel incarceration, and strangulation. We present a rare case of an adult patient presenting with an acute abdomen caused by small bowel strangulation due to a congenital transmesenteric defect. A 67-year-old female without any significant past medical or surgical history presented to the emergency department with an acute-onset worsening abdominal pain with nausea and vomiting. Physical examination revealed pain out of proportion to palpation of the abdomen. CT demonstrated internal hernia with moderate-volume ascites, concerning for ischemic bowel. The patient underwent an exploratory laparotomy, which demonstrated an internal hernia through a congenital mesenteric defect in the right hemiabdomen near the root of the mesentery with ischemic small bowel requiring resection. The patient was left in intestinal discontinuity and was returned for reexploration of the abdomen, open cholecystectomy, and right hemicolectomy. The patient’s recovery and hospital course were uncomplicated, and she was discharged from the hospital on postoperative day five. The incidence of congenital mesenteric defects in adults is exceedingly low, resulting in a limited body of literature addressing their clinical presentation and complications. Timely and accurate preoperative diagnosis is critical to prevent acute obstruction and strangulation of the bowel. This case contributes valuable insights into the diagnosis and management of this uncommon condition.

## Introduction

An internal hernia occurs when intestinal viscera pass through a natural or unnatural opening within the peritoneal cavity. This may lead to bowel obstruction, incarceration, or strangulation. These defects are either congenital or acquired. Acquired defects may be iatrogenic after surgical intervention (most commonly paraduodenal) or due to blunt abdominal trauma. Congenital transmesenteric hernias occur when any length of bowel herniates through an abnormal defect in the mesentery of the small bowel or the colon [1]. Congenital mesenteric defects can lead to a small bowel obstruction. This disease is more common in the pediatric population, with only 0.2% to 0.9% reported in adults [2]. These defects have been reported to be 2-3 cm in diameter. Patients often present with nonspecific findings such as intermittent abdominal pain, nausea, and vomiting. Rarely, an internal hernia can present as an acute abdomen secondary to intestinal obstruction, which has a mortality rate of up to 50%. Preoperative diagnosis remains challenging due to the nonspecific clinical presentation of the disease and the difficulty in diagnosis with CT imaging, which may identify the obstruction but miss the defect [2-4]. We present a rare case of a patient presenting with an acute abdomen caused by small bowel strangulation due to a congenital transmesenteric defect.

## Case presentation

**Figure 1:**
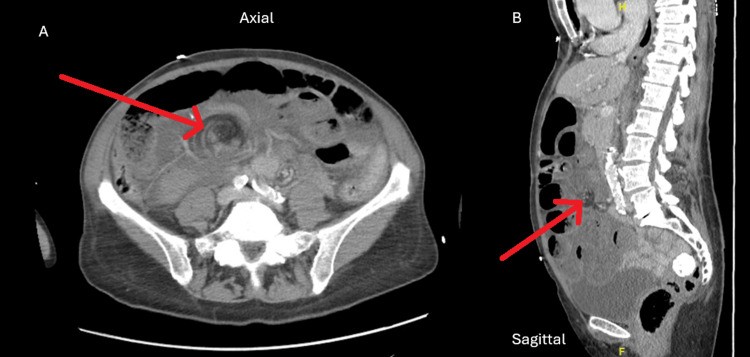
CT findings demonstrating a positive swirl sign with obstructive findings including a dilated small bowel. The red arrow demonstrates the swirl sign. A: Axial plane. B: Sagittal plane.

A 67-year-old female without any significant past medical or surgical history presented to the emergency department with an acute-onset worsening abdominal pain with associated nausea and vomiting. Physical examination demonstrated diffuse abdominal tenderness, sharp and severe in intensity, with guarding, suggestive of peritonitis. Laboratory findings were significant for lactic acidosis (lactic acid = 3.4 mmol/L, normal range = 0.7-2.0 mmol/L) and leukocytosis (white blood cell count = 15.7 k/mm^3^, normal range = 4.2-11.2 k/mm^3^). CT findings demonstrated an internal hernia with dilated loops of bowel and moderate-volume ascites concerning for ischemic bowel and swirling of the mesentery with convergence around a point in the right central abdomen around these loops (Figure 1).

The patient was started on volume resuscitation with lactated Ringer boluses. Empirical broad-spectrum antibiotics were initiated, and the patient was taken emergently to the operating room for an exploratory laparotomy. A nasogastric tube, a Foley catheter, an internal jugular central line, and arterial lines were placed in the operating room for close hemodynamic monitoring.

**Figure 2:**
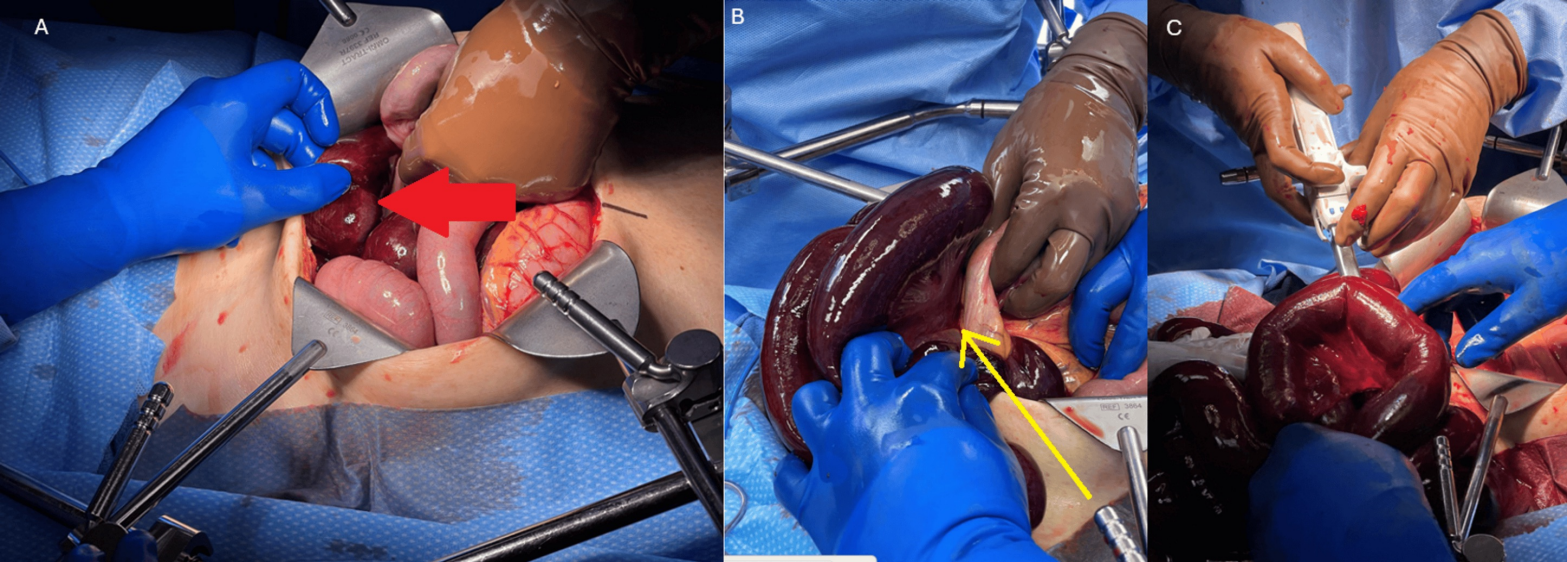
A: Intraoperative finding of an ischemic bowel with congested dilated loops. The red arrow demonstrates the transition between healthy and congested bowel. B: Identification of the mesenteric defect. The yellow arrow demonstrates the mesenteric defect. C: Application of the stapler for the removal of the ischemic bowel.

A midline laparotomy incision was made. Upon entry into the abdomen, hemorrhagic ascites was encountered along with grossly ischemic small bowel. The small bowel was run from the ligament of Treitz until the first point of obstruction in the internal hernia at the mid-jejunum. Distal to the obstruction, there was less than 10 cm of intact terminal ileum (Figure 2). The mesenteric defect, which was causing internal hernia, was dissected until the entire internal hernia could be reduced. The proximal and distal margins of healthy small bowel were selected, and the bowel was transected in the mid-jejunum and then at the terminal ileum (Figure 2) for a total of 290 cm of bowel resected. Greater than 300 cm of small bowel remained.

The patient was left in intestinal discontinuity with the intention for a second-look laparotomy to reevaluate the remnant bowel for any signs of ischemia the following day. The following day, the patient was hemodynamically stable for a second-look laparotomy. She was taken to the operating room with a plan to reestablish intestinal continuity. A spy indocyanine green exam was performed intraoperatively to determine the viability of the small bowel. This demonstrated a viable, well-perfused small bowel proximally, with questionable perfusion at the distal end of the terminal ileum. Due to the short length of the remaining ileum and vascular compromise of the ileocolic pedicle, a safe tension-free jejunoileal anastomosis would not have been a safe choice; hence, the decision was made to proceed with a formal right hemicolectomy. This was then followed by a stapled side-to-side jejunal colonic anastomosis. The mesenteric defect was then closed using a 3-0 silk suture in a running fashion. The rest of the abdomen was surveyed, and the gallbladder appeared largely distended with signs concerning for acute acalculous cholecystitis. The decision was made to proceed with an open cholecystectomy. The abdomen was then closed in layers, and the patient returned to the intensive care unit (ICU). The patient’s recovery and subsequent hospital course were uncomplicated. The patient was extubated in the operating room without complication and sent to the ICU. She was initially managed with nasogastric tube decompression, and after the return of bowel function on postoperative day three, she was started on a clear liquid diet. This was advanced to a regular diet. The patient was discharged from the hospital on postoperative day five.

The patient was followed up in the clinic postoperatively and was recovering appropriately without complications. Final pathology demonstrated benign small bowel mucosa and wall with marked ischemic change.

## Discussion

Due to the relative rarity of its presentation in adults, there is limited literature discussing congenital mesenteric defects in adults and the associated complications. Internal herniation of the bowel can lead to a variable degree of vascular compromise to the herniated bowel, which may present with nonspecific findings of abdominal discomfort or lead to acute intestinal obstruction [5]. This requires appropriate and timely preoperative diagnosis and emergent surgical intervention. Reported cases within the literature demonstrate that, similar to our case, all diagnoses of mesenteric defects were identified intraoperatively. These defects are most frequently found in the distal ileum [6]. It has been reported that approximately 30% of cases remain asymptomatic throughout life, which leads to this hernia only accounting for 5-10% of all internal hernias [7].

The pathogenesis of congenital mesenteric defects is often not identified. It has been hypothesized that these defects occur due to the disruption of the embryological steps involved in small bowel development. This includes failure of mesenteric fusion, abnormal resorption, vascular anomalies, and persistence of mesenteric windows [4]. Failure of mesenteric fusion can lead to fenestrations in the mesentery, leading to full defects over time. Abnormal resorption occurs with apoptotic changes within the mesenteric cells, leading to small defects that can grow. Vascular anomalies occur due to abnormal superior mesenteric artery development, leading to areas of mesentery without blood supply [8].

Butterworth et al. examined 13 case reports within the literature demonstrating bowel obstruction secondary to congenital mesenteric defects [2], of which four progressed to bowel ischemia. Clinical presentation varied from nausea and vomiting to severe abdominal pain and septic shock. A CT scan was only utilized preoperatively in four of the cases. In this case, CT findings were a supporting indicator of operative intervention; hence, preoperative CT can aid decision-making and assist in preoperative planning [2,9-12].

When establishing a differential diagnosis for obstruction in adults without an obvious external hernia or previous abdominal surgery, it is important to consider internal hernias secondary to congenital mesenteric defects. This facilitates prompt surgical intervention, including hernia reduction, resection of nonviable bowel, anastomosis, and defect closure. It is essential to preserve the mesenteric vasculature during closure of the defect. Previous case reports involve laying the small bowel along the length of the ascending colon and tacking it in place with sero-musculature sutures to broaden the root of the mesentery and prevent the small bowel from volvulizing [13].

## Conclusions

This case presents a unique perspective into the vague presentation and clinical challenges of diagnosis in patients with internal hernias secondary to congenital mesenteric defects. This case further expands on the unique pathology with involvement of the ileum and requirement of a right hemicolectomy and jejunal colonic anastomosis due to the limited length for a small bowel anastomosis. As more cases of internal hernias without significant surgical or traumatic history are reported, this case report demonstrates the importance of an expeditious workup for patients with congenital mesenteric defects and the prompt operative management required to help decrease morbidity and mortality.
